# An Efficient and Chemistry Independent Analysis to Quantify Resistive and Capacitive Loss Contributions to Battery Degradation

**DOI:** 10.1038/s41598-019-42583-2

**Published:** 2019-04-29

**Authors:** S. Bharathraj, S. P. Adiga, R. S. Patil, K. S. Mayya, T. Song, Y. Sung

**Affiliations:** 1 Materials and Simulations Group (SAIT-India), Samsung R&D Institute India-Bangalore, Bangalore, India; 2 Energy Materials Lab, SAIT, Samsung Electronics, Suwon, Republic of Korea

**Keywords:** Batteries, Batteries

## Abstract

Degradation mechanisms leading to deterioration in the battery performance is an inevitable phenomenon. Although there are detailed physics and equivalent circuit based models to predict the losses incurred due to degradation in estimating the health of the battery, they are either incomplete, computationally expensive or both. In this study, we present a very simple and elegant, chemistry independent mathematical analysis, which accurately calculates resistive and capacitive components of cycle-life related losses in a battery system. We demonstrate that discharge profiles obtained at any given degradation state of the battery can be represented by an analytical function, with its origin lying at the heart of battery dynamics, using simple parameter fitting. The model parameters relate to the battery electrochemical potential, resistance and capacity. We first validate our protocol using simulated cycling data from a degrading lithium-ion battery system modeled with detailed electrochemical thermal calculations and show that the estimates of capacity and power fades are >99% accurate using our method. Further, we construct a unique phase space plot of normalized energy, power that gives a compact representation of quantitative and qualitative trend of the degradation state of the system, as well as available power and energy.

## Introduction

The increasing demand for clean and rich sources of renewable energy has made employing electrochemical energy storage very attractive in applications ranging from power grids to electric vehicles^1^. The vast application space and substantial socio-economic and environmental implication has placed the electrochemical battery based energy storage systems at the forefront of energy research^2^. One of the key challenges facing wide-spread adoption of battery technology is the propensity of electrochemical cells to degrade and hence limiting their ability to provide acceptable levels of power and store/dispense adequate amount of energy as required, as the cells age. In addition to the potentially huge expense to replace the battery, the uncertainty associated with degradation dependent power and energy capability can dissuade faster adoption of the battery technology^3^. Therefore, the knowledge of cell state of health (SOH) and the ability to quantify its effect on battery capability becomes crucial in accurately predicting cell state of charge (SOC) and defining cell performance for different operating conditions to design well-informed control algorithms in battery management systems (BMS).

State of health (SOH) is often used to quantify the extent of degradation, which includes capacity and power fade. While the main electrochemical reactions enabling charge storage are reversible, there are irreversible parasitic reactions in the system leading to the deterioration of battery performance^4,5^. The intertwining of the effects of different side reactions are complex in nature and the ageing/degradation in the system through capacity/power fading, cannot be attributed to a single mechanism nor can they be studied independent of each other. For example, in the case of lithium-ion batteries (LIB), the formation of an immobilized layer of inactive material on the electrode surface known as SEI (Solid Electrolyte Interphase) causes increased internal resistance leading to power as well as capacity fade^6,7^. Whereas dissolution of the transition metal of the cathode material in acidic electrolytes, leads primarily to capacity fade^8–10^. These and other mechanisms can influence both power and capacity fade as they interact with each other.

Build-up of inactive materials on the active material surface of the electrodes, clogging of the migration channels of the ions/electrons cause resistance increase in the system. This manifests as a vertical drop in the discharge profile (Voltage Vs Time) as the term “IR” (current times resistance) contributes negatively (during discharge) and positively (during charge) in the overall cell voltage^11^. This is considered as a reversible loss, as running the system at very low values of current necessarily eliminates this contribution. Another type of degradation is the loss of the active material (AM) hosting the active lithium or the loss of cyclable lithium itself (loss of lithium inventory(LLI)) leading to the overall capacity reduction of the cell^4^. This manifests as a shrinkage on the time axis of the discharge curve. This type of loss unlike the previous one, is irreversible, as the lost active material is not recoverable. Capacity fade can also be due to the resistive loss^12^. This is because of the operational constraints put on the system. A drop in the discharge voltage due to resistance, leads to the profile reaching the minimum voltage cut-off faster during discharge, thus manifesting as a shrinkage on the capacity/time axis. As mentioned, this is a recoverable loss due to its reversible nature, in general.

Thus, reliable operation of a battery necessitates accurate determination of the degradation mechanism and quantification of the losses in an efficient but simple manner. Among current approaches, detailed physics based models, incorporating all the relevant electrochemical processes in the cell are capable of predicting battery degradation states accurately. These protocols take into account all the conservation equations, leading to a system of partial differential equations, which are numerically solved on platforms like MATLAB, COMSOL to obtain detailed profiles. Conservation equations of mass, charge and energy along with their relevant initial and boundary conditions are solved using numerical schemes, given the geometrical, operational and other system parameters. These calculations, referred to as electro-chemical-thermal models (ECT), are based on the Pseudo Two Dimensional (P2D) model, first developed by Newmann’s group^13^. More recently, ECT models have been extended to account for side reactions that lead to resistive and capacitive degradation mechanisms^14^. In this case, the aging mechanisms are studied in detail and their influence on the system is mathematically modelled and incorporated in the conservation equations. For example, the anode undergoes degradation due to the formation of the solid-electrolyte-interphase as mentioned before. The SEI formation^6,7^ on the anode side has been modelled using Tafel kinetics, which consumes a part of the applied current, the magnitude of which depends on the over-potential of the reaction. Thus, the charge conservation equation will have an additional ‘sink’ term to account for this side reaction. Due to the resistance offered by the formed SEI layer, there is a voltage drop proportional to this resistance, and thus a capacity fade as well. These mechanisms are well captured by the detailed ECT models as detailed in later sections, especially in the supplementary information and cited references. While ECT based models are capable of describing degradations accurately, they are computationally very expensive and are accurate to the extent that one has incorporated all relevant degradation mechanisms in a representative manner. The computational expense of these detailed models is somehow mitigated through what are called reduced order models (ROM)^15,16^ where significant reduction in computational time is achieved by approximating partial differential equations in the ECT model and condensing the detailed physics through, either ordinary differential equations or linear algebraic expressions, via volume averaging. In modelling degradation, however, they suffer from the inability to capture spatially dependent degradation dynamics and hence perform poorly for cells with thick electrodes or high C-rate operating conditions. Apart from these physics based models, there are other methods based on estimating the SOC/SOH of the system using simpler algorithms^17^, that lack the accuracy of ECT based models. For example, the equivalent circuit model (ECM) based techniques rely on representing the cell through a series of circuits with resistors and capacitors. The Thevenin model, even though is simple and extensively used, it can only be used to explain the temporary responses of the battery at given SOCs. The charge discharge management, relationship between the SOC and OCV etc. cannot be described using these models. The model parameter identification to suit the particular ECM is a cumbersome process as well^18^. Also, there are non-invasive and non-destructive techniques like the electrochemical impedance spectroscopy (EIS)^1,19,20^, which bring into light the ageing mechanisms in a cell. The method is dependent on analyzing the Nyquist plots with frequencies, which are signatures of the different geometric attributes and reactions/processes in the system. This protocol also has its own drawbacks as it is not suitable for on-board applications. Also, processes which have very similar time scales (which is highly likely), cannot be distinguished on the frequency plots. All these observations point towards the need for a simpler but complete protocol, which can quantify different types of degradation in the system.

In the current study, we present a simple and a powerful protocol to represent a discharge profile of a battery system capturing its salient features, which when compared with a fresh cell (or an earlier degradation state), is able to predict the degradation state, resolving capacitive and resistive losses. Because of the operating conditions and system thermodynamics, we recognize the open circuit potential (OCP) and hence the discharge profile in a battery to have a logarithmic variation of voltage vs time^21^. We utilize this concept and represent the discharge profile using an asymmetric sigmoidal function with a linear perturbation, whose coefficients are capable of representing the physics of the system in a minimal yet accurate way. The coefficients also lead to the prediction of the losses individually, thereby estimating the SOC, SOH, also the available power in the system, simultaneously. We also introduce a novel way to analyze degradation, whereby the normalized power and capacity of a battery are plotted as a function of ageing. These plots, quantitatively and qualitatively predict the type of degradation, the losses incurred and the shifts between resistive and capacity losses over cycling, giving a holistic view of the status of the system.

## Results and Discussions

The cell voltage is determined primarily by the difference in chemical potential between anode and cathode, i. e. the potential at which the electrodes exchange Li^+^, which in turn gets modified by cell internal resistance which itself is dependent on operational parameters. The thermodynamic connect between chemical potentials and lithium contents of anode and cathode is given by the change in internal potential energy per intercalated Li^+^. This quantity is dependent on the extent of lithiation^21^ as determined by the shape of Gibbs free energy of the solid solution of Li and the electrode material. Since in a cell, one electrode is lithiating when the other is delithiating, the resulting open circuit voltage (OCV) of the cell is the difference of open circuit potentials of individual electrodes that typically has an asymmetric sigmoidal behavior. We utilize this knowledge to express the cell characteristic profiles like discharge/charge profile and the OCV in mathematical terms. The analytical form chosen for this model is an asymmetric sigmoidal function with a linear term:

1 $$y=\frac{c}{(1+ax{e}^{bx})}+dx$$

**Figure 1:**
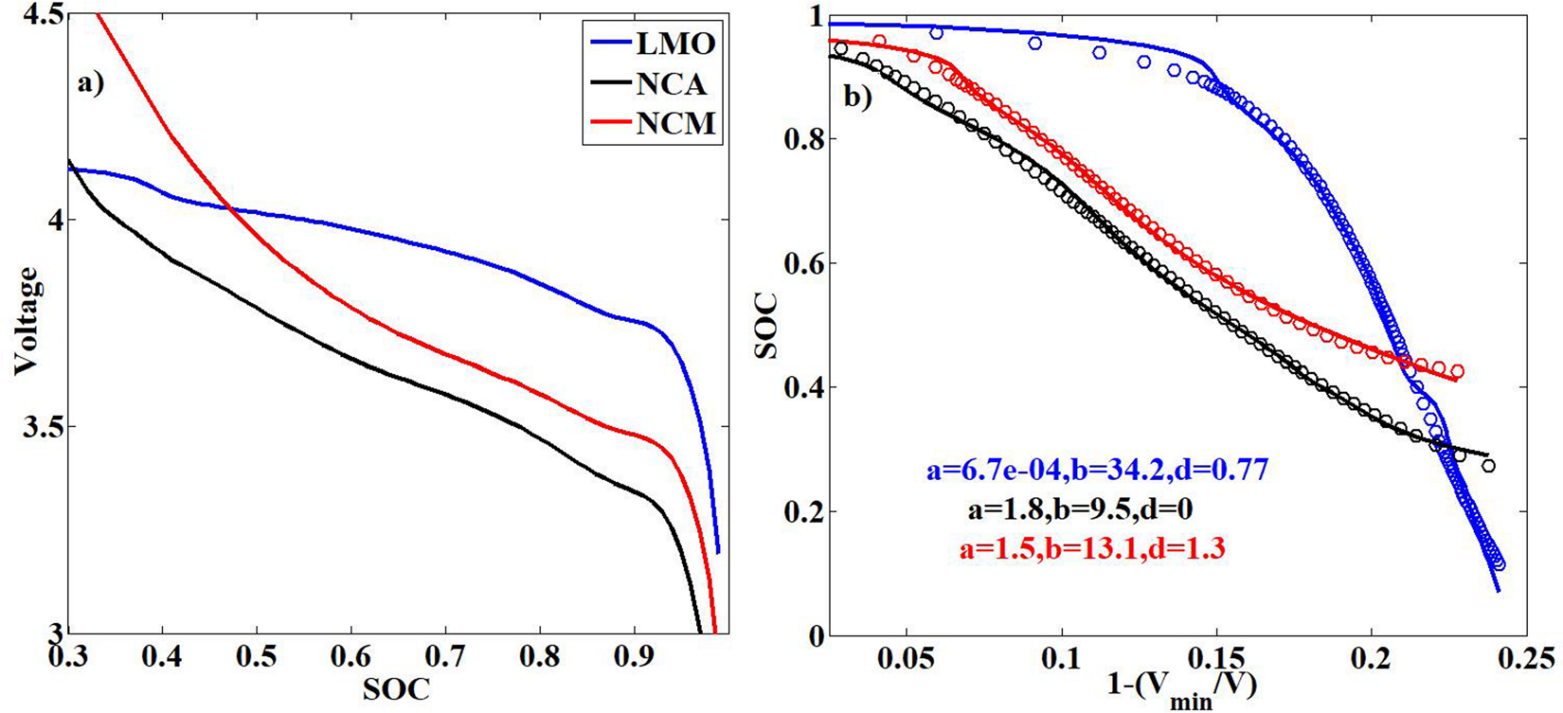
Proof of concept of the model using commonly used Battery chemistry OCVs. (**a**) The OCVs of cells with NG as anode with different cathode chemistries. (**b**) The OCV data rescaled as per the model description (solid lines) and the prediction of the profiles by the model (circles).

Where, *y* is the time, *x* is the normalized voltage, with *a, b, c* and *d* as the model parameters. We first establish that this analytical form is able to represent OCVs of most common electrode materials. The OCVs of cells with NG as anode and cathode chemistries like LMO (Lithium Manganese Oxide), NCA (Nickel Cobalt Aluminum Oxide) and NCM (Nickel Cobalt Manganese Oxide), are represented through this model as shown in Fig. 1. In the model representation, the axes of the discharge (could very well be the charge) or the OCV profile, as the case may be, is reversed. The time/SOC is presented on the y-axis and the normalized voltage given by,

$1-\frac{{V}_{min}}{V}$, is presented on the x-axis. *V*_*min*_ is the voltage cut-off at the end of discharge. In Fig. 1(a), the OCVs as a function of SOC is shown for the different chemistries (SOC > 0.2). In Fig. 1(b), they are represented in a modified form, where the SOC is on the y-axis and the normalized voltage on the x-axis. The model as shown in Eq. (1), is applied to these curves, with the parameter *c* being 1. As seen, the model is able to capture the salient features of the OCVs with excellent accuracy. The fits have an R^2^ value of >0.99.

It is quite easy to see that the model parameters represent the system characteristics as shown in Fig. 1(b). For example, c is a direct measure of the SOC_max_(which is 1 in all the cases) or the capacity of the system in the case of a discharge profile. This is because the battery capacity,$\,Q={\int }_{t=0}^{t={t}_{disch}}Idt=I{\int }_{t=0}^{t={t}_{disch}}dt=I{t}_{disch}$, where, ${t}_{disch}$, is the time taken for full discharge and *I*, is the constant current input. It is clear that $c={t}_{disch}$ as y = c at x = 0 (or V = V_min_). The parameter, *b*, is a measure of the curvature of the profile, thus is a characteristic of the concerned battery chemistry and the C-rate, with the parameter, *a*, being the normalization factor. The linear term is added to the sigmoidal function as a means to curtail the asymptotic behavior of the sigmoidal function at the cutoffs. The losses incurred due to degradation is estimated as follows.

As a direct measure of the capacity loss in the system, the parameter c is estimated and the difference with respect to the value at the first cycle, c_0,_ gives the total capacity loss. To get a measure of the resistive loss, the voltage at the start of discharge, at a given discharge rate, is calculated and the difference with the first cycle (fresh cell), V_0,_ gives the actual measure of this loss. This is obtained from the model by extracting the value of voltage at time, *y* = 0. Rearranging the model for *y* = 0 gives the form:

2 $$ad{x}^{2}{e}^{bx}+dx+c=0$$

which is solved for x numerically. An analytical expression can be obtained by solving the above equation, but the expression lacks simplicity and elegance. Moreover, with the current optimization algorithms available, the given Eq. (2) can be solved with an initial guess for *x*. Thus, the model gives the exact measure of both the capacity and resistive losses at the same time, alongside system characteristics like available power and energy. The area under the curve of a discharge profile gives a measure of the total energy in the system. This area, when divided by the total time, gives the measure of the mean power derivable from the system. Energy and power for a particular discharge cycle are defined as:

3 $$E={\int }_{t=0}^{t={t}_{disch}}Vdt$$

4 $$P=\frac{{\int }_{t=0}^{t={t}_{disch}}Vdt}{{t}_{disch}}=\frac{E}{{t}_{disch}}$$

*E*_0_, *P*_0_, are the energy and power respectively of the fresh cell. *V* is the voltage and *t*_*disch*_ is the total time for discharge. We utilize the concepts related to Eqs (3 and 4), in greater detail for the analysis in the last part of this section.

To check the efficacy of the mathematical model, it is important to test it under the different degradation scenarios leading to capacity and/or resistive fade. Thus, we test the accuracy of model prediction against simulated cell ageing with different combinations of active degradation mechanisms. The cell ageing simulations are done using detailed electrochemical thermal models that include degradation reactions. To elicit resistive loss in the system, we invoke the SEI resistance model^3^ for the anode and the Manganese dissolution model^22–24^ for capacity fade. These two models are used in different combinations (3 of them) to get different types and extents of degradation. The system, models involved and the degradation mechanisms, are explained in brief in the methods section. Also, the model parameters, its values, related to the system, operational conditions and degradation models are given in the Table 1–4 in the methods section. We also validate the model against the NASA battery experimental degradation data^25^.

**Table 1:** List of parameters used in the simulations.

Parameters	Anode	Separator	Cathode
	NG		LMO
Thickness, (μm)	50	25	60
Solid ftaction *ε*_*s*_	0.563		0.629
Liquid fraction *ε*_*l*_	0.401	0.43	0.271
Particle radius, *r*_*p*_ (*μm*)	14.9 × 10^−6^		2.4 × 10^−6^
*c*_*s*._max (*mol/m*^3^)	28600		23339
*D*_*s*_ (*m*^2^/s)	1.4523 × 10^−13^		1.0 × 10^−14^
*k*_*r*_ (m/s)	3.11 × 10^−11^		2.00 × 10^−11^
Active material Fraction	1.0		1.0
*SOC*_*min*_	0.05		0.35
*SOC*_*max*_	0.90		0.995
*c*_*e*_ (*mol*/*m*^3^)	1150	1150	1150

**Table 2:** Operating conditions used in the simulations.

Charge and Discharge windows	Value
*CC* charge cut off voltage, *V*_*max*_	4.1 V
*CV* charge cut off current, *I*_*min*_	0.01 1 C current
*CC* discharge cut off voltage, *V*_*min*_	3.7 V

**Table 3:** Parameters used for the Anode-SEI growth model.

SEI film growth parameters	Value
Equilibrium potention, *U*_*SEI*_	0.38 V
Molecular weght	$\,0.10\frac{{\boldsymbol{kg}}}{{\boldsymbol{mol}}}$
Density	$2100\frac{{\boldsymbol{kg}}}{{{\boldsymbol{m}}}^{3}}$
Exchange current density, ${{\boldsymbol{i}}}_{{\boldsymbol{SEI}}}^{0}$	$8.6\times {10}^{-8}\frac{{\boldsymbol{A}}}{{{\boldsymbol{m}}}^{2}}$
Electronic conductivity	$3.79\times {10}^{-7}\frac{{\boldsymbol{S}}}{{\boldsymbol{m}}}$
*Charge transfer coefficient*, *α*_*SEI*_	0.5

**Table 4:** Parameters used for the LMO dissolution model.

LMO dissolution model parameters	Value
Equilibrium potential, *U*_*side*_	4.1 V
Specfic area times Exchange current density, ${\boldsymbol{a}}\,{{\boldsymbol{i}}}_{{\boldsymbol{side}}}^{0}$	$10\frac{{\boldsymbol{A}}}{{{\boldsymbol{m}}}^{3}}$
Rate constant, *K*_2_	7.3 × 10^−10^ *m*^6^*mol*^−2^*s*^−1^
Rate constant, *K*_3_	2 × 10^−10^*ms*^−1^
Charge transfer coefficient, *α*_side_	0.5
molar volme of LMO, $\bar{{\boldsymbol{V}}}$	4.1389 × 10^−5^*m*^3^*mol*^−1^

The intent here is to mathematically model the characteristic cell profiles (discharge profile) and be able to capture the physics of the system through its parameters. The proof of concept for this model was elaborated through Fig. 1. Now, we exercise the same protocol on discharge profiles obtained from simulations with different models inducing different types of degradation. To show case the efficacy of the protocol under different scenarios, we have chosen one specific battery chemistry with its relevant degradation models, and analyzed them for different combinations of the same. The system chosen for analysis is a Lithium-Ion Battery (LIB) cell with NG-LMO electrodes. The Anode-SEI model^3^, which leads to the formation of a passive layer of inactive substances leading to impedance increase, is included for the anode side. For the cathode side, we incorporate the Manganese dissolution model^26^, whereby parasitic side reactions of solvent and electrolyte decompositions lead to the production of protons. The acid thus generated, attacks the cathode active material leading to the dissolution of Manganese. This causes reduction in the active material volume fraction, as well as loss of lithium inventory, leading to capacity fade in the system. Further details are available in the supplementary information and in the cited works. The geometric, transport and other kinetic parameters used for the simulations, operating conditions of the system, parameters related to the Anode-SEI model and LMO dissolution model are given in the methods section. The complete system is simulated through the ECT models developed in-house at Samsung Advanced Institute of Technology. The detailed description of this model can be found in other works^27^ and is not pertinent to the scope of the present work. The discharge curves obtained from the ECT simulations, the capacity and resistive losses from both the simulations, model predictions are analyzed.

### Simulations with LMO dissolution model

**Figure 2:**
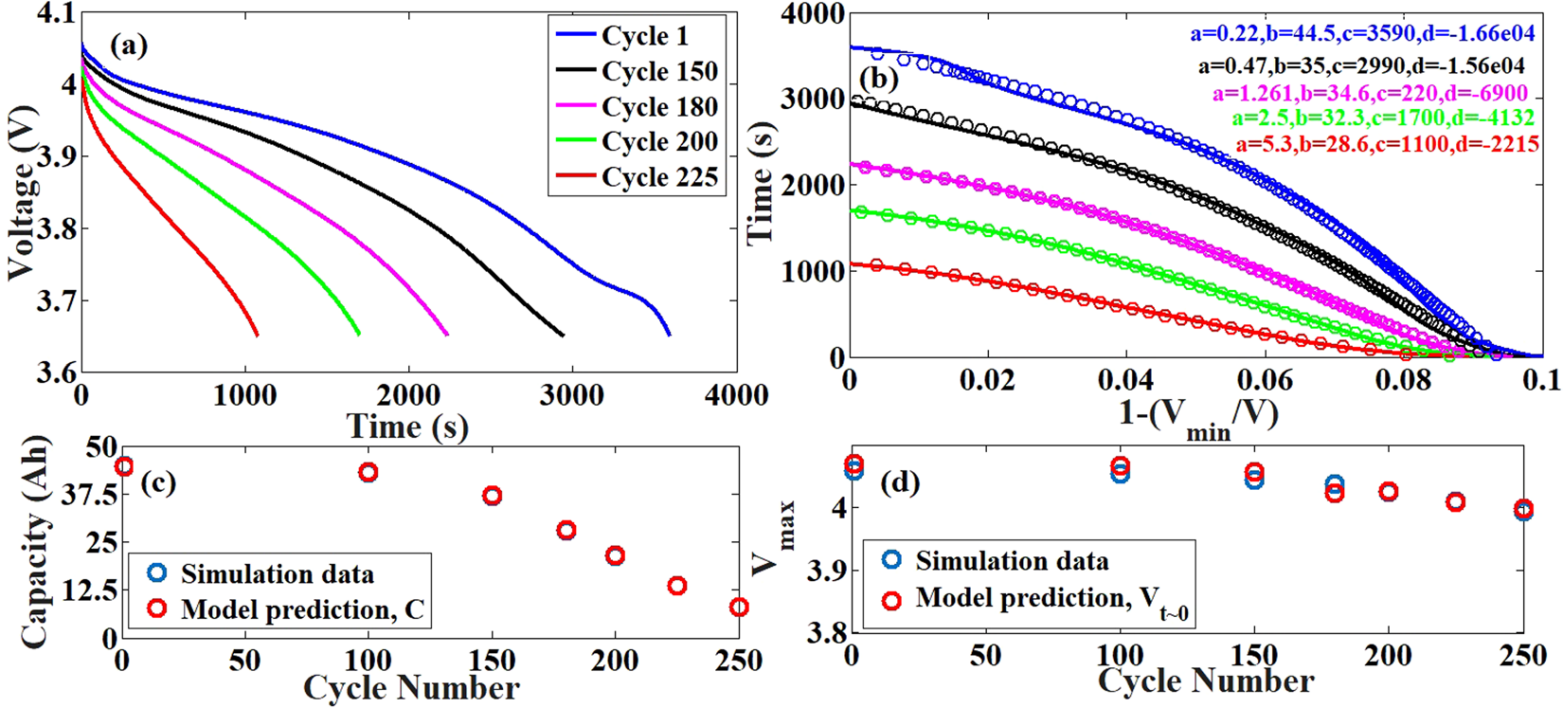
Comparison between simulation data and model prediction for the NG-LMO system with only LMO degradation. (**a**) The discharge profiles obtained from ECT simulations using the LMO dissolution model. (**b**) The reduced parameter representation of the discharge curves in the new system (bold lines) and the model prediction (circles) with the parameter values mentioned in the legend. (**c**) The comparison of the capacity loss (available capacity) over cycling obtained from the ECT simulations and the prediction from the model. The time taken for full discharge over a cycle is taken as a measure of the capacity. (**d**) The comparison of the resistance induced loss obtained from the simulations and the prediction from the model. The voltage at the start of discharge (V_max_) is taken as a measure to predict the resistance induced loss. The maximum error between the predicted and actual data is <0.4%.

In this system, as we have imposed only the cathode degradation model, we expect only capacity fade in the system due to AM and LLI. It is a complex network of reactions (details in the supplementary section and in the cited papers) inducing degradation in the system. Although the transport coefficients do decrease with decrease in the active material volume fraction, thus inducing internal resistance, the impedance rise is too small to be of any concern. The model parameters are such that the system experiences almost 50% capacity fade at the end of 200 cycles as shown in Fig. 2(a). We do not see any significant decrease in the voltage at the beginning of discharge as seen in Fig. 2(c), as expected. Our model is able to capture both the small amount of resistance build up and the significant capacity fade experienced, accuracy greater than 99%.

### Simulations with the Anode SEI model

**Figure 3:**
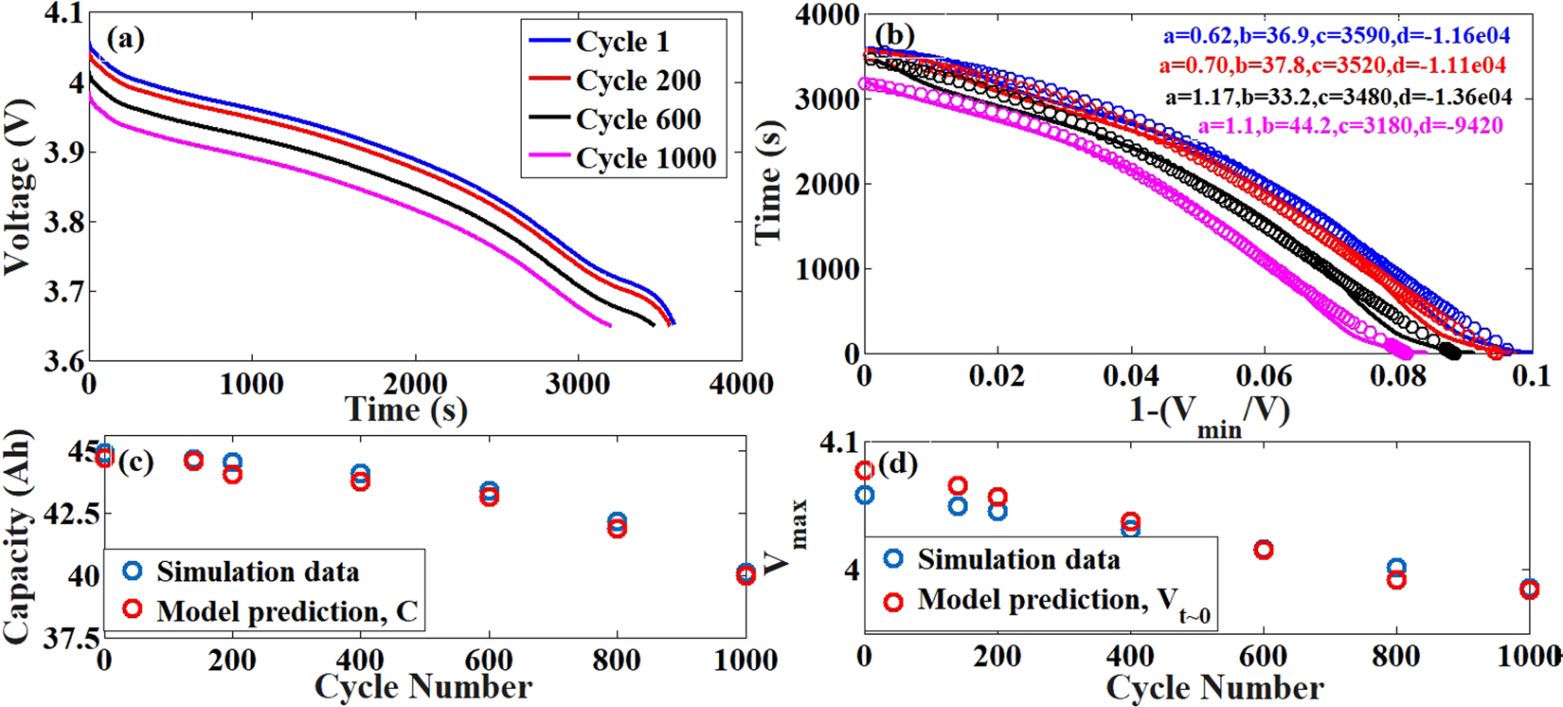
Comparison between simulation data and model prediction for the NG-LMO system with only the Anode SEI model. (**a**) The discharge profiles obtained from ECT simulations using the Anode SEI model. (**b**) The reduced parameter representation of the discharge curves in the new system (bold lines) and the model prediction (circles) with the parameter values mentioned in the legend. (**c**) The comparison of the capacity loss (available capacity) obtained from the simulations and the prediction from the model. The time taken for full discharge over a cycle is taken as a measure of the capacity. (**d**) The comparison of the resistance induced loss obtained from the simulations and the prediction from the model. The voltage at the start of discharge (V_max_) is taken as a measure to predict the resistance induced loss. The maximum error between the predicted and actual data is <0.5%.

In the Anode SEI model, there is a build up of an inactive porous layer called the SEI, on the active material particles of the anode. The slow growth of this passive layer results in the accumulation of resistance, inducing losses in the system. The discharge curves obtained with this model is as shown in Fig. 3(a). The resistance fade and thus the induced capacity fade are clearly visible over cycling due to the SEI formation. As seen, in Fig. 3(c,d) respectively, the model is able to predict both the resistive and capacity losses in the system with accuracy >99%.

### Simulations with both the degradation models

**Figure 4:**
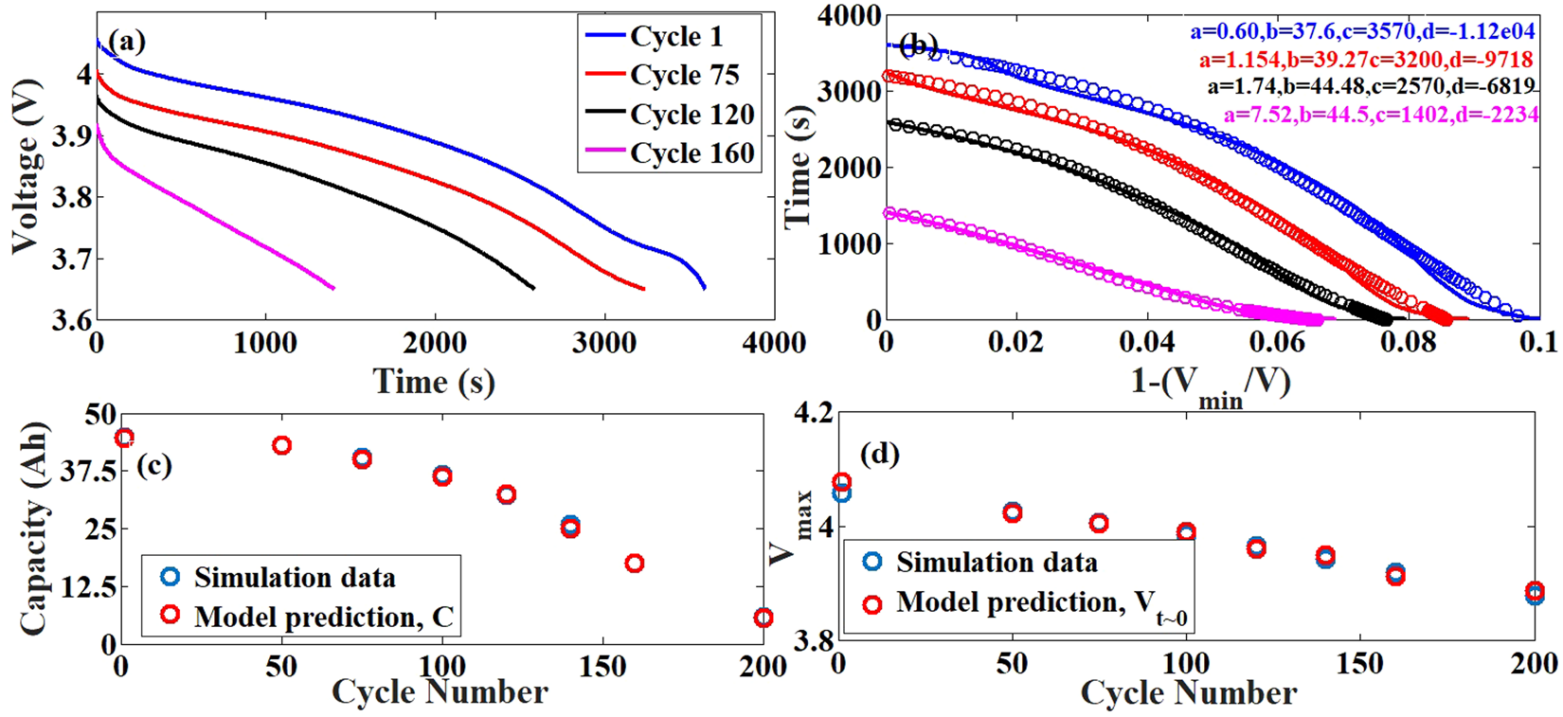
Comparison between simulation data and model prediction for the NG-LMO system with both Anode SEI and LMO degradation. (**a**) The discharge profiles obtained from ECT simulations using the Anode SEI and LMO dissolution model. (**b**) The reduced parameter representation of the discharge curves in the new system (bold lines) and the model prediction (circles) with the parameter values mentioned in the legend. (**c**) The comparison of the capacity loss (through available capacity) obtained from the simulations and the prediction from the model. The time taken for full discharge over a cycle is taken as a measure of the capacity. (**d**) The comparison of the resistance induced loss obtained from the simulations and the prediction from the model. The voltage at the start of discharge (V_max_) is taken as a measure to predict the resistance induced loss. The maximum error between the predicted and actual data is <0.5%.

In this study, both the Anode SEI and LMO dissolution models are included. As both these mechanisms induce degradation in the system in more or less independent ways, we expect to see a combined effect of the same on the losses. The resistive loss induced capacity fade gets added up with the capacity fade induced due to the active material loss on the cathode side. As seen in Fig. 4(a), there is both resistive and capacity loss in the system. The capacity loss is much more pronounced as compared to the system with only LMO degradation mechanism.

As seen in Fig. 4(b,c), our model is able to capture both the resistive and capacity losses with great accuracy. Although in a realistic scenario, operating beyond a capacity fade of 60% itself is a rarity, this exercise was done to prove the efficacy of the model even at very extreme limits of degradation. The data shown till now are all simulation generated, physics based model induced data. The next set of results focusses on the protocol being exercised on an experimental data set.

### Verification with experimental data

Now we apply our protocol to analyze experimental Li-ion battery ageing, where cycling data available from NASA’S Li ion aging data repository^25^ has been used. More details of the dataset can be found on the data repository page. In this data set the cathode is NCA as opposed to LMO used in our ECT simulations. Also, the operational window and system geometry are totally different as compared to the simulations. Thus, it will be of interest to see the efficacy of the model.

**Figure 5:**
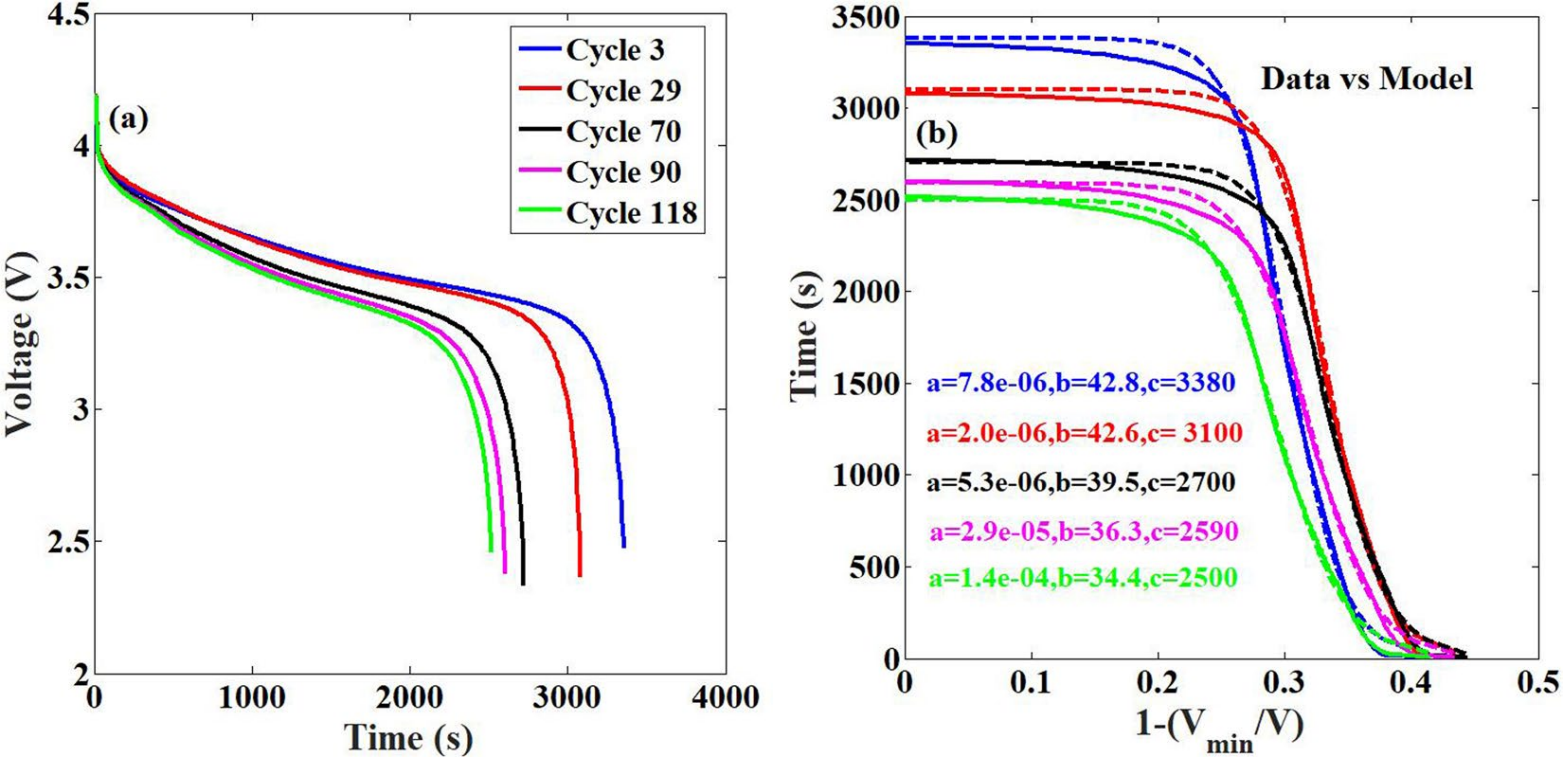
Comparison between simulation data and model prediction for the experimental data on Li-ion battery ageing from the NASA repository^25^. (**a**) The discharge profiles obtained from NASA repository [19] (The file number used is B0018). (**b**) The discharge data rescaled as per the model description (solid lines) and the prediction of the discharge profiles by the model (circles) clearly indicating the presence of only capacity loss in the system. The model parameters are as shown in the legend. The maximum error between the predicted and actual data is <1%. The value of the parameter ‘d’ is zero in the case which corresponds to the NCA chemistry, as shown in Fig. 1(b).

The discharge profiles extracted from the dataset are as shown in Fig. 5(a). A firsthand analysis shows that the system shows only capacity fade. The model prediction of the discharge profile is shown in Fig. 5(b). The model is able to capture the nuances of the discharge profiles in great detail and accuracy.

These results prove that our protocol is able to capture the nuances of battery performance characteristics like the OCV, discharge profiles with a simple mathematical model. Because of the aptness, accuracy of the proposed function in the manuscript, model equations and its predictions on ageing, the battery characteristics especially the degradation trends are now known in real time. Both the energy (capacity) and power (voltage) losses are independently predicted for the user to take corrective action. For example, in the case of an LMO cathode, as the capacity fade characteristics are known in advance, charge-discharge protocols can be designed in such a way, as to limit the time spent in the high voltage regime (where cathode dissolution is maximum) and thus the degradation. This helps in prolonging the cycle life of the battery. This is a significant leap in the direction of battery technology advancement and adoption, as it leads to enhanced control over the battery prognostics.

### Representation of battery degradation characteristics

Typically, the power and energy capabilities of electrochemical energy storage systems are represented using Ragone plots^28^ that show the relationship between energy and power density on a log-log plot. By presenting energy and power densities, either gravimetric/volumetric, we analyze how operating the battery at low/high power changes the energy one can derive from the system. Moreover, as new battery chemistries are introduced, plotting Ragone charts allows for benchmarking electrodes/devices against existing systems. Here, we extend this concept to provide graphical representation of degradation characteristics of a battery system with clarity on power and capacity fade as compared to a fresh cell. We use average power and capacity of an aged battery and normalize these with respect to corresponding values for a fresh cell. The fresh cell values of average power and capacity used for normalization are obtained at a discharge rate of 1 C. To represent degradation characteristics, the normalized power is plotted against normalized capacity. In this representation, as cells degrade, a shift along the x-axis relates to change in range (energy/time/capacity) and a shift along the y-axis represents change in power capability. Thus, a purely resistive degradation is expected to lead to reduction in the average power and a purely capacitive degradation is expected to result in reduction of the range for a given power.

As seen from the definitions in Eqs (3 and 4), energy contains the information of both the dimensions of voltage and time (and thus resistive and capacity loss information) and power is the mean voltage of the system. If there are no resistive losses, the mean normalized power, $(\frac{P}{{P}_{0}})$, will remain the same with respect to the normalized energy $(\frac{E}{{E}_{0}})$ or capacity $(\frac{c}{{c}_{0}})$. The difference in the abscissas of the plots between normalized power $(\frac{P}{{P}_{0}})$ Vs normalized capacity $(\frac{c}{{c}_{0}})$ and normalized power $(\frac{P}{{P}_{0}})$ Vs normalized energy $(\frac{E}{{E}_{0}})$ curves will, thus give a measure of the contribution due to “IR” loss (resistive loss). Thus, a horizontal line in the normalized power Vs (vs) normalized capacity/energy plot indicates a system with capacity loss due to active material loss. A vertical line in this phase space plot is not possible, as a system with resistive losses, will inevitably induce capacity losses (recoverable) as explained earlier. Thus, any deviation from active material loss induced degradation (capacity fade indicated horizontal line) in the anti-clock wise direction indicates resistance induced degradation or a combination of both.

**Figure 6:**
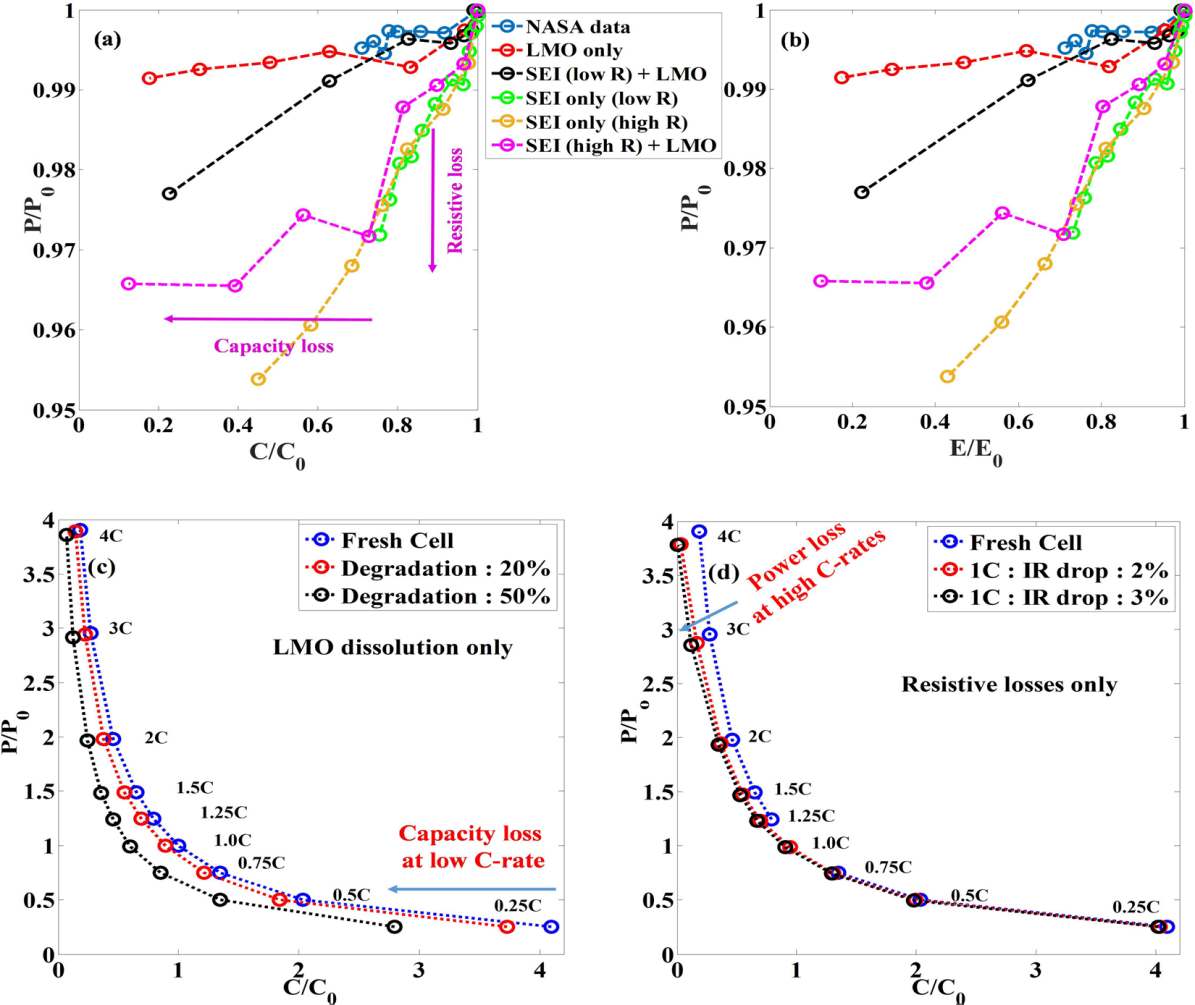
Representation of degradation characteristics of ageing cells in the power-energy-capacity space. (**a**) The normalized power as a function of the normalized time for systems with different degradation mechanisms. (**b**) The normalized power as a function of the normalized energy for systems with different degradation mechanisms. Plot showing the normalized power as a function of the normalized time for different degraded states at different C-rates for different combinations of degradation mechanisms (**c**) LMO dissolution only and (**d**) Anode SEI only (with different resistance values). Normalization is with respect to the corresponding value at 1C of the fresh cell.

Figure 6 summarizes the above method for all the different systems we analyzed in the previous section. We see that systems which have purely capacity fade and no impedance rise (Figs 2 and 5) have almost horizontally flat profiles showcasing only energy/capacity fade. On the contrary, systems with predominantly impedance rise (Fig. 3), showcase a profile with steep slope. Depending on the dominant mechanism, we see profiles with different slopes tending towards capacity-fade-only systems to resistance-fade-only systems, with combinations of both in between.

We also observe systems transiting from one mechanism to the other as seen in the system with both SEI (high R) and LMO dissolution. In Fig. 6(a,b), one can see the shift in the degradation mechanism, whereby after a large resistance induced power fade, the system transits into energy fade showcasing the capacity loss. Thus any clock-wise transitions point towards more capacity fade and anti-clock wise ones lead to impedance rise/power drop. Interestingly, any difference between the normalized energy and capacity abscissas for a given power state, give the loss due to resistance drop. This is quite intuitive as energy includes losses due to both resistance and capacity fade.

This protocol is a very powerful technique in delineating between the different degradation mechanisms in a simple yet effective manner. In addition, it is able to capture the transitions between the different mechanisms over cycling with cyclic shifts in the profile. Moreover very relevant information like the available power and energy from the system, which is of utmost priority in battery management systems (BMS) can be extracted.

Figure 6(c,d) shows a contour of the power-energy phase space for cells (at different levels of degradation) with respect to different C-rates. This analysis aims at understanding the effect of degradation on extracting useful power and energy from systems at different C-rates, which have undergone different extents of degradation. For a system, which has undergone capacity fade due to AM/LLI (irrecoverable), the energy/time axis will show the effect at very low C-rates as a full measure of the available capacity is obtained. This is clearly evident from Fig. 6(c). At a C-rate of 0.25, we clearly see the difference in the capacities for the different degraded states, which is reduced at higher c-rates. In addition, we do not see any loss of available power from any of the degraded states with respect to the fresh cell, as there are no resistance induced losses in the system. Although there is a slight decrease in the power, observed at very high C-rates. This is due to the effect of large currents on transport properties at high C-rates leading to a small internal resistance in the cell. In Fig. 6(d), we see the opposite. As capacity loss induced is through the resistance increase, the capacity is recoverable as the loss is essentially eliminated by operating the system at very low C-rates. For a C-rate of 0.25, there is no difference in the capacities for the different degraded states. At the same time, we see a significant loss of power, which becomes more prominent at high C-rates. The resistive and capacity losses have their own signatures in the power-energy space. This kind of analysis, can be extended for other degradation mechanisms and their combinations as well, yielding very useful information regarding the performance characteristics and the type of deterioration involved.

## Conclusions

In this study, we presented and analyzed, detailed electrochemical model simulations using degradation models to generate profiles with different types and extents of degradation. Models like the Anode-SEI and LMO-dissolution were utilized in different proportions to induce losses to different extents. We discussed a novel protocol, where by the inherent battery characteristics like the OCV and performance characteristics like discharge profiles are modeled using an analytical expression rooted in battery dynamics. The parameters of the analytical expression provide an accurate estimate of the resistive and capacity fades in the system simultaneously, without any computational cost. They also reveal additional performance characteristics like available power and energy at any degradation state. The efficacy of this protocol was shown through studies on the results generated through detailed ECT simulations of an NG-LMO battery. Irrespective of the degradation model used or the battery chemistry, we were able to capture the nuances of the characteristic cell profiles and the degradation spectra with excellent accuracy, which was also proved using an experimental dataset.

We then introduced a new graphical representation to depict degradation characteristics as the cell ages by plotting normalized power vs normalized capacity. This representation reveals information regarding how resistive and capacity fades manifest as one cycles a battery, and its ramifications with respect to available power and energy in the system. Any shifts in the mechanism of degradation is captured by the transition in the clock-wise or anti clock-wise direction in this phase space. Analysis of the influence of discharge rates on degraded states was also carried out. This reveals very interesting and useful information regarding the available power and energy from the system, given the extent of deterioration. When used along with a battery management system, this analysis can help change the degradation course of a battery by tweaking the appropriate operational parameters.

## Methods

### Computational

The results generated and analyzed in this work are computational in nature. The simulations, as described earlier, were performed on an in-house ECT code written at Samsung Advanced Institute of Technology. The ECT model had both the Anode SEI^3–7^ and LMO degradation^22–24,26^ models incorporated in them. Depending on the type of simulations, the relevant modules were turned on/off. More details regarding the model are available in the supplementary information and in the cited papers. The NG-LMO system parameters, operating conditions, parameters for the SEI model and LMO dissolution model are given in the following tables.

## Supplementary information

SUPPLEMENTARY INFOR

## Data Availability

All data generated or analyzed during this study are included in this published article (and its Supplementary Information files). If any additional information is required, they are available from the corresponding author on reasonable request.
