# Supplementary material for: An Efficient and Chemistry Independent Analysis to Quantify Resistive and Capacitive Loss Contributions to Battery Degradation
Source: Sci Rep. 2019 Apr 29;9:6576. doi: 10.1038/s41598-019-42583-2 (PMC6488653; doi:10.1038/s41598-019-42583-2)
Supplement: Supplementary file 1 — SUPPLEMENTARY INFOR [file 41598_2019_42583_MOESM1_ESM.docx]

**An efficient and chemistry independent analysis to quantify resistive and capacitive loss contributions to battery degradation**

S. Bharathraj^1,^*, S. P. Adiga^1,^*, R. S. Patil^1^, K. S. Mayya ^1^, T. Song^2^, Y. Sung^2^

^1^Materials and Simulations Group (SAIT-India), Samsung R&D Institute India-Bangalore, India.

^2^Energy Materials Lab, SAIT, Samsung Electronics, Republic of Korea.

* Corresponding Authors. E-mail: [s.bharathraj@samsung.com](mailto:s.bharathraj@samsung.com) (S. Bharathraj); [shashi.adiga@samsung.com](mailto:shashi.adiga@samsung.com) (S. P. Adiga)

**Supplementary Information**

**Summary of the degradation models used in the NG-LMO system**

To the Anode-SEI model, the solvent (Ethylene Carbonate mostly) undergoes reduction at the interface of the anode-electrolyte during the charging process (at a potential of ~ 0.38V) to precipitate an insoluble layer on the anode surface as shown in equation (A.1). This layer, which is called the solid-electrolyte-interface (SEI) offers resistance to ionic movement. This reaction is modeled by a cathode Tafel kinetics expression as shown in equation (A.2), with the over potential of the reaction being described by equation (A.3). The total current applied to the system is a combination of the current required for the intercalation reaction and the contribution towards the formation of the SEI layer as shown in equation (A.4). The over potential for the intercalation reaction, will now have a contribution due to the SEI layer formation in the form of a resistance drop, offered by the SEI layer as shown in equation (A.5).

$Solvent+{Li}^{+}{+ e}^{-}\to$ ${Product}_{SEI} (A.1)$

$$i_{SEI}=-i_{SEI}^{0} exp\left( -\frac{\alpha_{SEI}F}{RT}\eta_{SEI} \right) (A.2)$$

$$\eta_{SEI}=\phi_{s}-\phi_{e}-U_{SEI}-iR_{SEI} (A.3)$$

$$i=i_{int}+i_{SEI} (A.4)$$

$$\eta=\phi_{s}-\phi_{e}-E_{eq}-IR_{SEI} (A.5)$$

Although the SEI layer seems to be an unwelcome participant in the system, it is a blessing in disguise. The formation of this layer prevents further exposure of the active material to more degradation, thus acting as a protective cover. As this layer formation is due to consumption of the active material (which is irreversible) and offers resistance to the ionic movement, this process leads to both capacity and resistive losses respectively.

For the cathode side, we incorporate the Manganese dissolution model, whereby parasitic side reactions of solvent and electrolyte decompositions lead to the production of protons. The acid thus generated, attacks the cathode active material leading to the dissolution of Manganese and thereby reducing the active material volume fraction, leading to capacity fade in the system.

The solvent decomposition leading to the acid formation is as shown in equation (A.6). The solvent decomposition is described by an anode Tafel kinetics expression with the over potential containing the side reaction equilibrium potential, *U_side_* in the equations (A.7) & (A.8) respectively. Unlike equation (A.3), it is assumed that the insoluble products formed offer zero resistance. The rate of acid generated is given by equation (9). The total current applied to the system, as shown in equation (A.4), will now have an additional contribution due to (7) as well, if the cathode dissolution model is also switched on. The over potential is also, accordingly modified.

$Solvent\to$ $Insoluble products+H^{+}{+ e}^{-} (A.6)$

$$i_{side}=i_{side}^{0} exp\left( \frac{\alpha_{side}F}{RT}\eta_{side} \right) (A.7)$$

$$\eta_{side}=\phi_{s}-\phi_{e}-U_{side} (A.8)$$

$$R_{s1}= \frac{i_{side}}{F} (A.9)$$

The second side reaction leading to the production of protons, is a two-step electrolyte decomposition as shown in equation (10). The progress of this reaction depends on the concentration of residual water in the system. Unlike the previous reaction, this reaction, is not controlled by charge transfer kinetics and thus has a kinetic expression of the form given in equation (11). As the ionization potential of $LiPF_{6}$ is very high, the concentration of the same can be approximated to be that of ${Li}_{+}.$

LiPF_6_  🡪 LiF + PF_5_
 *(A.*10*)*
 PF_5_ + H_2_O 🡪 POF_3_ + 2HF

$$R_{s2} = K_{2}[H_{2}O]^{2}[LiPF_{6}] \approx K_{2}[H_{2}O]^{2}\left[ {Li}_{+} \right] (A.11)$$

The acid generated due to the above side reactions, (A.6) & (A.10), attack the cathode active material (LMO) to cause Manganese dissolution according to the equation (A.12). The reaction is observed to be acid dominated and thus the kinetic expression in equation (A.13).

4H^+^ + 2LiMn_2_O_4_ 🡪 2Li^+^ + Mn^2+^ + (3/2) Mn_2_O_4_ + 2H_2_O (*A*.12)

$$R_{s3} = K_{3}\left[ H^{+} \right] (A.13)$$

$$\frac{\partial\varepsilon_{pos}}{\partial t}= -a_{pos}R_{s3}\bar{V} (A.14)$$

As the above sequence of reactions involve the generation of new species like H_2_O, H^+^ and Mn^2+^ , the mass balance equations are accordingly written for the concerned species. Due to the acid induced dissolution, there is a loss of active material, whose governing equation is as given in equation (A.14). This explains the capacity loss incurred in the system on the cathode side.
